# Broadband Terahertz Fingerprint Metasurfaces via Localized Surface Plasmon Resonance and Electromagnetically Induced Transparency Synergy

**DOI:** 10.3390/bios16100566

**Published:** 2026-10-09

**Authors:** Feng-Lei Guo, Hao-Yi Yang, Jiu-Sheng Li

**Affiliations:** 1 Centre for THz Research, China Jiliang University, Hangzhou 310018, China; tidegfl@cjlu.edu.cn (F.-L.G.); p24030854052@cjlu.edu.cn (H.-Y.Y.); 2 Zhejiang Key Laboratory of Optoelectronic Information Technology, Hangzhou 310018, China

**Keywords:** localized surface plasmon resonance, electromagnetically induced transparency, terahertz metasurface, biosensing

## Abstract

We developed a terahertz broadband sensing chip based on the synergistic enhancement effect of localized surface plasmon resonances (LSPRs) and electromagnetically induced transparency (EIT). This chip consists of a periodic array formed by cross-shaped metasurface elements of varying sizes and E-shaped metasurface elements, enabling broadband biochemical substance detection within the 0.50–2.50 THz frequency range while maintaining high sensitivity and broad spectral fingerprint resolution capability. Through analysis of electric field distribution, surface current distribution, and multipole decomposition theory, the physical mechanisms underlying near-field localization enhancement and far-field scattering regulation were systematically revealed. Experimental results demonstrate that the sensor achieves the lowest experimentally detected concentration of 5 μg/mL for lycopene. The characteristic peak sensitivities, derived from the envelope variation rate, are 10.44 × 10^−3^ a.u./(μg/mL) and 2.67 × 10^−3^ a.u./(μg/mL), respectively. This chip exhibits significant advantages in trace biochemical substance detection and broadband fingerprint recognition, offering new insights for the in-depth application of metasurface technology in the biosensing field.

## 1. Introduction

The metasurface composed of subwavelength resonant elements can generate extremely strong local electromagnetic field enhancement effects at the interface, greatly enhancing the interaction strength between terahertz waves and the analyte under test. In recent years, researchers have continuously explored various physical mechanisms to further improve the sensitivity of metasurface sensors [1,2]. For example, the anapole mode enhances near-field localization by suppressing far-field radiation scattering [3,4]. The EIT effect utilizes destructive interference between bright and dark modes to create a sharp transparent window in a wide absorption spectrum, significantly enhancing the field localization and dispersion characteristics near resonance [5,6]. Quasi-bound states (Q-BIC) [7,8] transform bound states in continuous spectra into resonant modes with extremely high-quality factors by breaking structural symmetry. Chen et al. [9] designed a double split-ring metasurface for detecting hyaluronic acid, with a frequency shift detection limit of 0.958 pmol/µL and a transmittance detection limit of 0.02 pmol/µL. Kutuzova et al. [7] designed a defective disk metasurface with a Q value of 319. Cai et al. [10] designed a water-shaped metasurface for detecting tartaric acid, with a sensitivity of 590 GHz/RIU and a detection limit of 1 mg/mL. In order to further improve sensitivity, metasurfaces are combined with microfluidic technology and nano-metal materials to further enhance detection performance. For example, Cao et al. [11] designed a notched cylindrical nano-gold metasurface with microfluidic design, achieving a sensitivity of 1.65 × 10^6^ NW/RIU. Liu et al. [12] designed a nano-gold metasurface for detecting miRNA-451 using multi-channel microfluidics, with a detection limit of 19.2 fM. However, the aforementioned metasurface can only detect substance content and lacks specific recognition ability. Therefore, researchers proposed using multiple resonant peaks to form a broadband metasurface sensor [13,14]. Xue et al. [15] studied the formation of envelope curves on four cylindrical metasurfaces through angular scanning, with a working range of 1.7 THz to 2.05 THz. The detection limits for azodicarbonamide, sorbic acid, and melamine were 1.65 µg cm^−2^, 1.20 µg cm^−2^, and 1.66 µg cm^−2^, respectively. Xu et al. [16] designed a triangular prism tetramer to detect cinnamoyl glycine by changing the polarization angle, with a working range of 0.43 THz to 0.56 THz, a detection limit of 1.23 µg cm^−2^, a Q value of 231, and an FOM of 609. Liu et al. [17] designed graphene metasurfaces for the upper-loop resonator (U-RCR) and lower-loop resonator (D-RCR), forming a detection curve in the 1.5 THz frequency band with a detection limit of 64 μg cm^−2^. Wang et al. [18] designed a double-gap square-frame metasurface based on Fano-type interference with a working frequency range of 1.15–2.69 THz and identified L-glutamic acid. An EIT-like response arises from the destructive interference between two discrete resonant modes (bright–dark mode coupling), forming a symmetric transparency window. In contrast, Fano resonance originates from the interference between a continuum state and a discrete resonant state, which yields an asymmetric spectral profile described by the Fano factor. Moreover, owing to the nonlinear optical field enhancement occurring in the Fermi-level tuning of graphene, envelope distortion arises, and a sophisticated “fingerprint spectral inversion model” is needed to restore the line shape. Furthermore, existing research mostly focuses on narrowband- or single-frequency-point-enhanced detection, making it difficult to cover multiple fingerprint characteristic peaks of biomolecules in the wide spectral range of terahertz frequency. The enhanced detection of broadband terahertz fingerprint spectra is of great significance for comprehensively obtaining molecular structural information and achieving precise identification of complex mixtures. The synergistic effect of localized surface plasmon resonance (LSPR) and the EIT effect deserves attention. LSPR can generate sub-wavelength-scale electric field localization on the surface of metal microstructures, thereby achieving enhanced detection of terahertz fingerprint signals, while the EIT effect can further compress the resonant linewidth and enhance the interaction time between light and matter. In summary, broadband metasurfaces not only enable substance recognition through fingerprint spectra, but also possess quantitative detection capabilities, demonstrating broad prospects in the field of terahertz biosensing. How to integrate the synergistic enhancement effect of LSPR and EIT on a single metasurface platform to achieve terahertz fingerprint spectral detection with both high sensitivity and broadband coverage is still a cutting-edge topic that urgently needs to be explored.

In this article, we propose a terahertz broadband metasurface fingerprint spectroscopy technique based on the synergistic enhancement of LSPR and EIT. By designing an integrated metasurface with 14 different subarray resonant elements, strong field localization via LSPR excitation is achieved while introducing the EIT effect to further compress the resonant linewidth and enhance terahertz wave–matter interactions, resulting in a broadband envelope curve covering the 0.50–2.50 THz range. The goal is to enable broadband, high-sensitivity detection of trace biomolecular fingerprints, validated through lycopene-based terahertz wave sensing. This study not only maintains stable characteristic fingerprint spectra but also achieves quantitative detection, balancing sensitivity with wide-spectrum fingerprint recognition capabilities in label-free detection. It provides new insights for the development of terahertz metasurface biosensing technology and holds promise for practical applications in early disease diagnosis, drug screening, and environmental monitoring. In contrast, the proposed metallic metasurface exhibits a linear response between transmission peak intensity and analyte concentration, allowing direct and reliable quantitative detection without complex post-processing algorithms, which eases integration with microfluidic technologies and point-of-care testing devices.

## 2. Device Structure and Design

**Figure 1:**
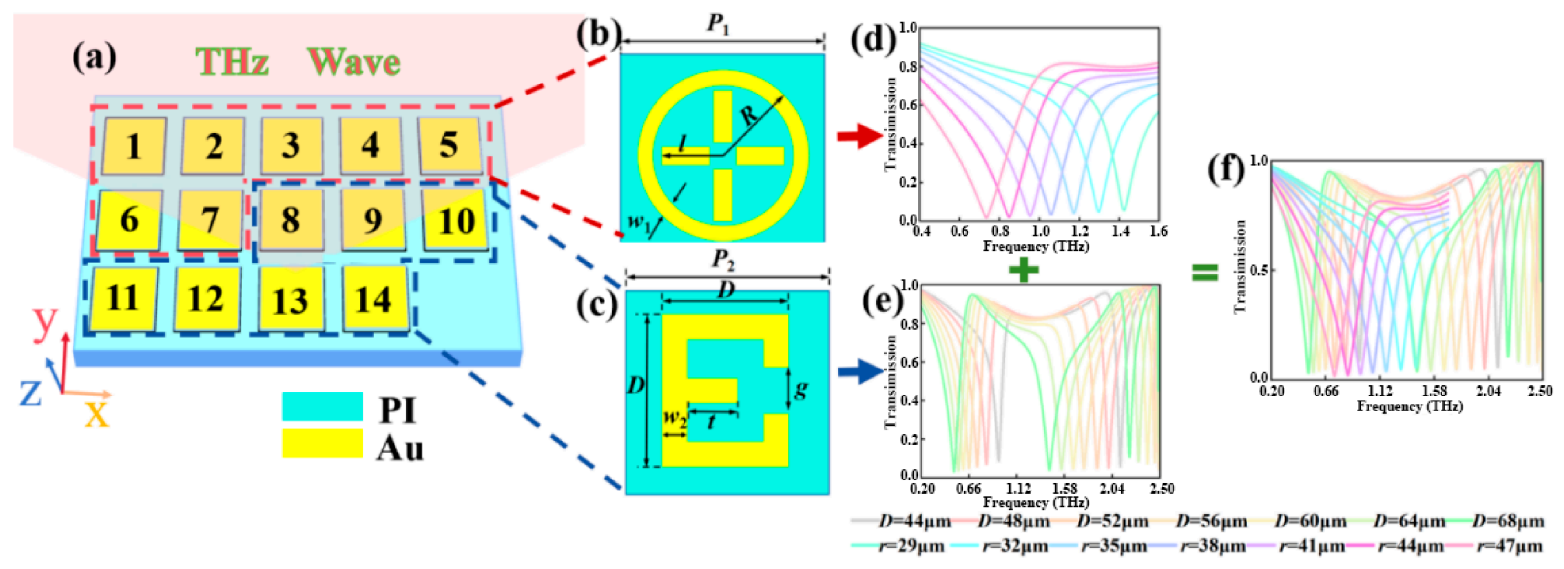
(**a**) Schematic diagram of dual-design metasurface terahertz sensing chip; (**b**) cross-shaped reticle metasurface element; (**c**) E-shaped metasurface element; (**d**) transmission spectra of cross-shaped reticle elements of different sizes; (**e**) transmission spectra of E-shaped elements of different sizes; (**f**) transmission spectra of sensing chip.

**Figure 2:**
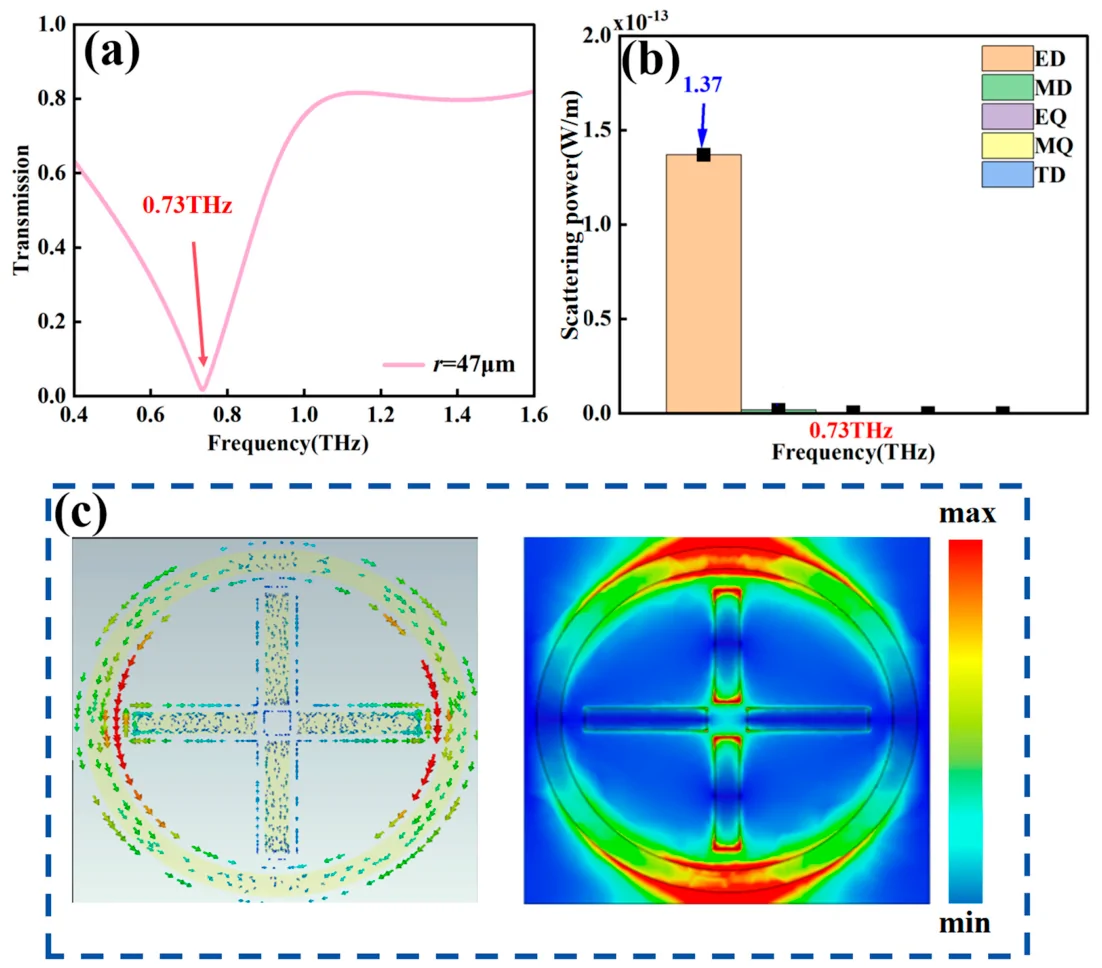
A cross-shaped quasi-star metasurface element with *r* = 47 µm. (**a**) Transmission spectrum; (**b**) surface current and electric field; (**c**) multipole scattering power.

The array-element-integrated metasurface terahertz sensing chip structure is shown in Figure 1a. Fourteen metasurface arrays are arranged on a polyimide (PI) substrate, with a relative dielectric constant of 3.5 and a thickness of 30 μm. The conductivity of the metal is 4.561 × 10^7^ S/m, and the thickness is 0.2 µm. The crosshair design is adopted in elements 1–7, and the E-shaped design is adopted in elements 8–14. The cross-shaped sight bead metasurface element with *P*_1_ = 100 µm and *w*_1_ = 6 µm is shown in Figure 1b. The center gap of the cross-shaped sight bead is 10 μm and *l* = *r*–12 µm (*r* = 47 µm, 44 µm, 41 µm, 38 µm, 35 µm, 32 µm, 29 µm). The E-shaped metasurface element is shown in Figure 1c with *P*_2_ = 80 μm, *w*_2_ = 10 μm, *g* = 16 μm, and *t* = *D*–29 μm (*D* = 68 µm, 64 µm, 60 µm, 56 µm, 52 µm, 48 µm, 44 µm). Figure 1d and Figure 1e depict the transmission spectra of seven different-sized cross-shaped sight bead arrays and E-shaped arrays, respectively. The theoretical detection frequency range of the sensing chip obtained by merging two transmission spectra is 0.50–2.50 THz, as shown in Figure 1f. In this article, we take the *r* = 47 µm cross-shaped sight bead metasurface element as an example and calculate the transmission spectrum of the metasurface element, as shown in Figure 2a, with a transmission peak at 0.73 THz. The distribution of the surface current and electric field is shown in Figure 2b. The electric field is strong at the junction of the circular ring and the vertical pole above and below the cross. The current flows downwards along the circular ring and upwards along the vertical pole, and the pattern is discontinuous, indicating that the resonance is caused by LSPR. By integrating the electromagnetic intensity in space, the scattering spectra of multipoles, including electric dipoles (EDs), magnetic dipoles (MDs), toroidal dipoles (TDs), electric quadripoles (EQs), and magnetic quadripoles (MQs), were calculated. The scattering power histograms of different multipoles at 0.73 THz are illustrated in Figure 2c. ED mainly contributes to the resonance peak, reaching 1.37 × 10^−13^ W/m.

**Figure 3:**
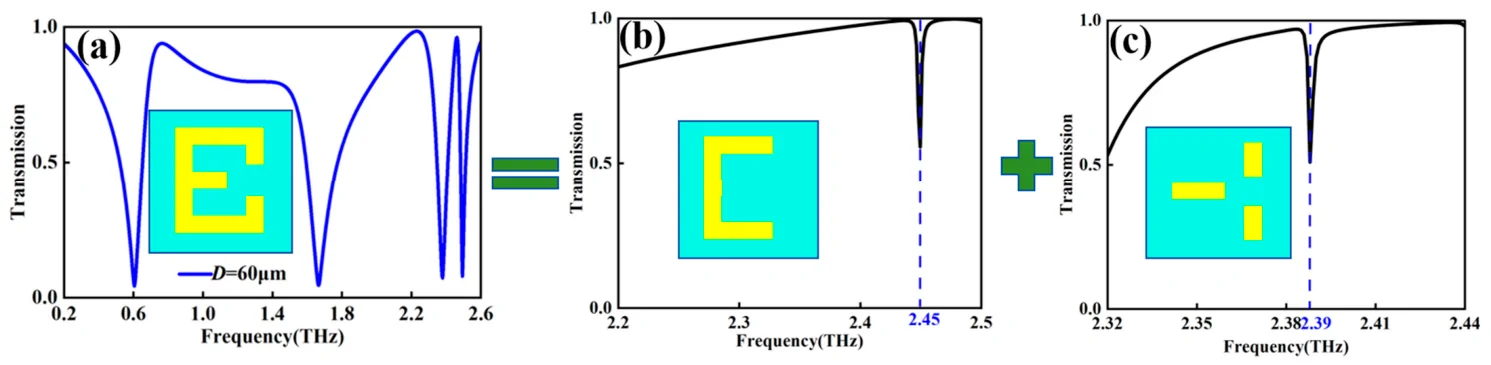
Transmission spectra of the E-shaped metasurface element and its decomposed resonator; (**a**) E-shaped metasurface; (**b**) c-shaped resonator; (**c**) three-rod resonator.

**Figure 4:**
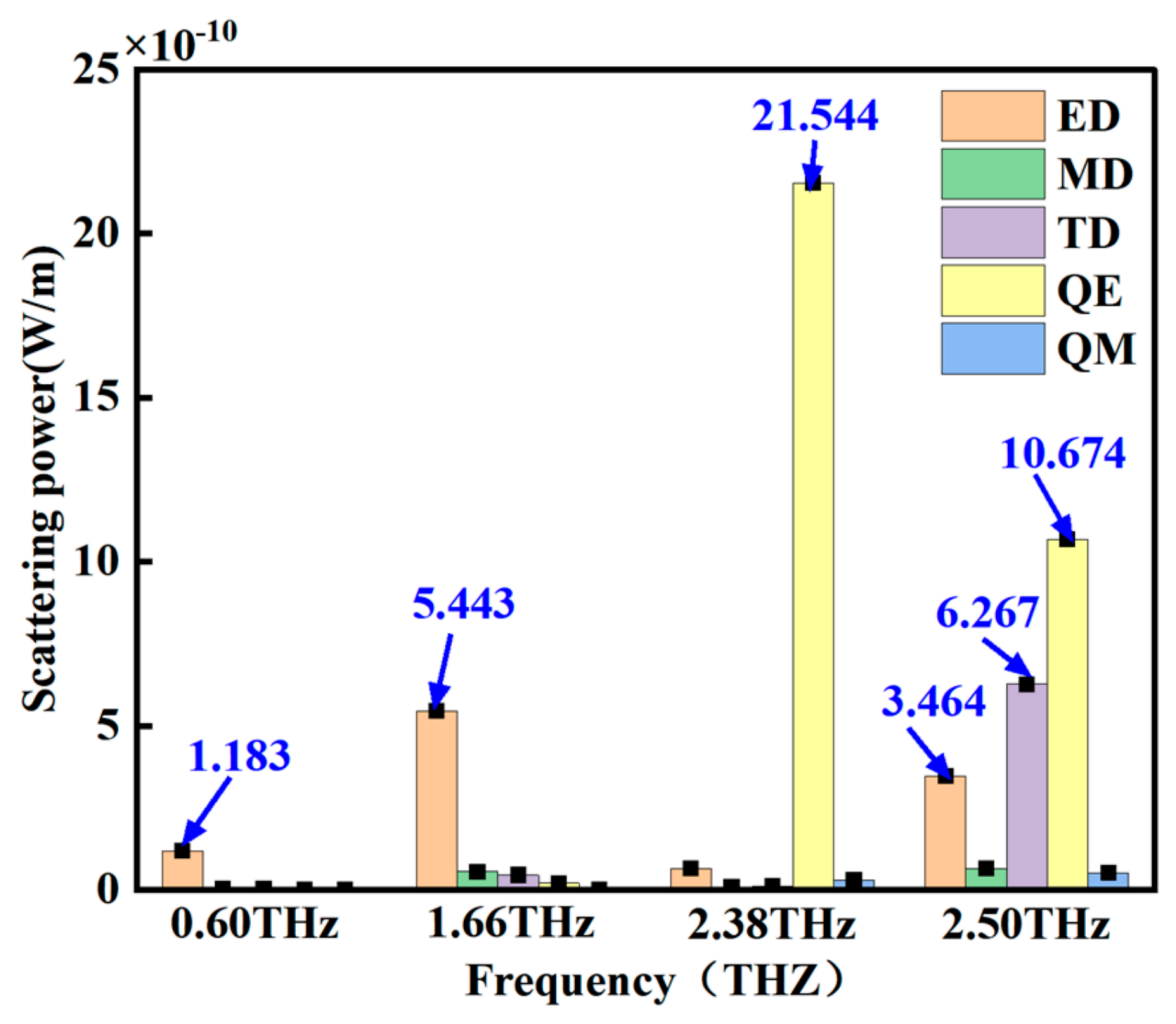
E-shaped metasurface element multipole scattering power diagram.

Taking the E-shaped metasurface element with *D* = 60 µm as an example, the transmission spectrum of the metasurface element was calculated and is shown in Figure 3a. There are four transmission peaks at frequencies of 0.60 THz, 1.66 THz, 2.38 THz, and 2.50 THz, and a transparent window appears at 2.46 THz. To explore the physical essence of the EIT effect, the transmission spectra of the substructures “C-shaped resonator” and “three-rod resonator” were calculated separately. The C-shaped resonator produces a resonance peak at 2.45 THz, as shown in Figure 3b. The three-rod resonator exhibits a resonance peak at a frequency of 2.39 THz, as given in Figure 3c. When these two substructures are combined, a transparent window appears at 2.46 THz due to the destructive interference of their scattering fields, indicating the occurrence of the EIT effect in the structure. The calculated scattering powers of different multipoles are shown in Figure 4. It can be seen that at frequencies of 0.60 THz and 1.66 THz, ED scattering is dominant, with powers of 1.183 × 10^−10^ W/m and 5.443 × 10^−10^ W/m, respectively. At a frequency of 2.38 THz, EQ scattering is most prominent, with a power of 2.154 × 10^−9^ W/m, and ED is suppressed. At a frequency of 2.50 THz, EQ, TD, and ED all contribute, with EQ scattering being the main source, reaching 1.067 × 10^−9^ W/m, followed by TD at 6.267 × 10^−10^ W/m, and ED at 3.464 × 10^−10^ W/m. It can be seen that ED dominates at lower frequencies, while EQ is the main scattering source at higher frequencies.

**Figure 5:**
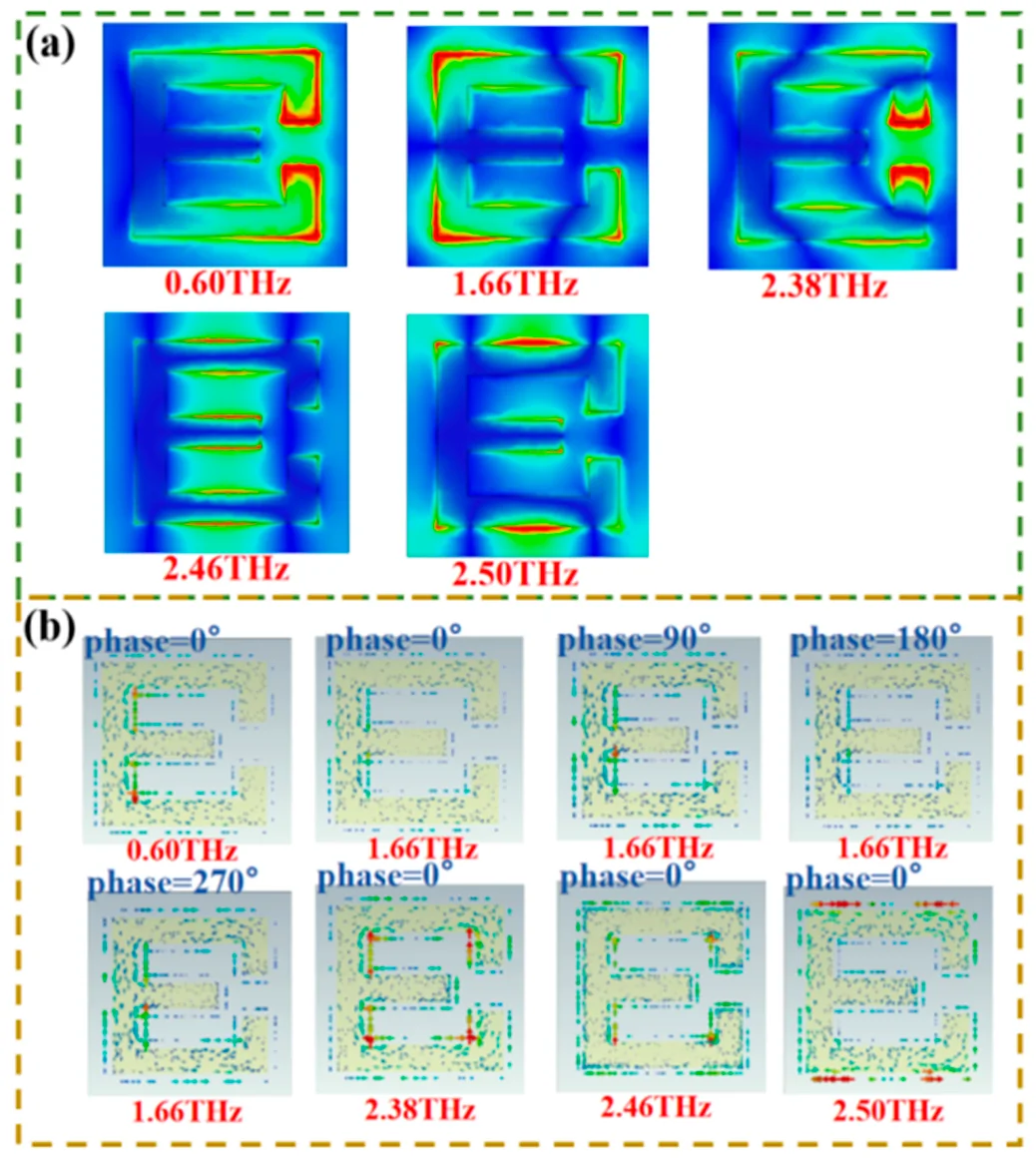
(**a**) Electric field diagram of E-shaped metasurface at different frequencies; (**b**) current diagram.

To further investigate the internal mechanism of terahertz wave–matter interactions in the near-field of E-shaped metasurfaces, the electric field and current distributions of E-shaped metasurface elements were calculated at different frequencies, as shown in Figure 5a,b. At a frequency of 0.60 THz, the electric field is concentrated in the gap between the two vertical rods on the right side, and the current flows downwards along the main body from above the gap without a reverse current. This is the ED scattering excited by LSPR, which is in bright mode. At a frequency of 1.66 THz, the electric field is symmetrical and strong at the outer angle. When the phase is 0°, the current in the left vertical pole is upward, the horizontal bar in the upper half structure is to the left, and the horizontal bar in the lower half structure is to the right, forming a charge distribution of negative in the upper left, negative in the lower right, positive in the upper right, and positive in the lower left, which is a typical characteristic of EQ. The presence of current vortices in the middle horizontal bar is an MD characteristic, which is in a quasi-dark mode (with non-zero radiation losses). At phases of 90° and 180°, the vertical pole current is downward, the upper half of the structural crossbar is to the right, and the lower half of the structural crossbar is to the left, with the positive *x*-axis direction as the observation direction. The current vortex inside the crossbar is clockwise. When the phase is 270°, the current flow direction is consistent with when the phase is 0°. This fixed directional correlation proves that synchronous coupling is formed between the quasi-dark mode and bright mode, but the scattering field does not completely interfere, so the EIT phenomenon does not occur. At a frequency of 2.46 THz, the electric field forms a symmetrical quadrupole moment, and the current reverses on the rod, with EQ as the bright mode. At 2.50 THz, the electric field is symmetrical on both sides, with the current flowing towards the center on the upper horizontal bar, outward on the lower horizontal bar, and upward on the vertical bar. There is a small vortex inside the middle horizontal bar, which is the result of the joint action of multiple poles.

## 3. Results and Discussion

**Figure 6:**
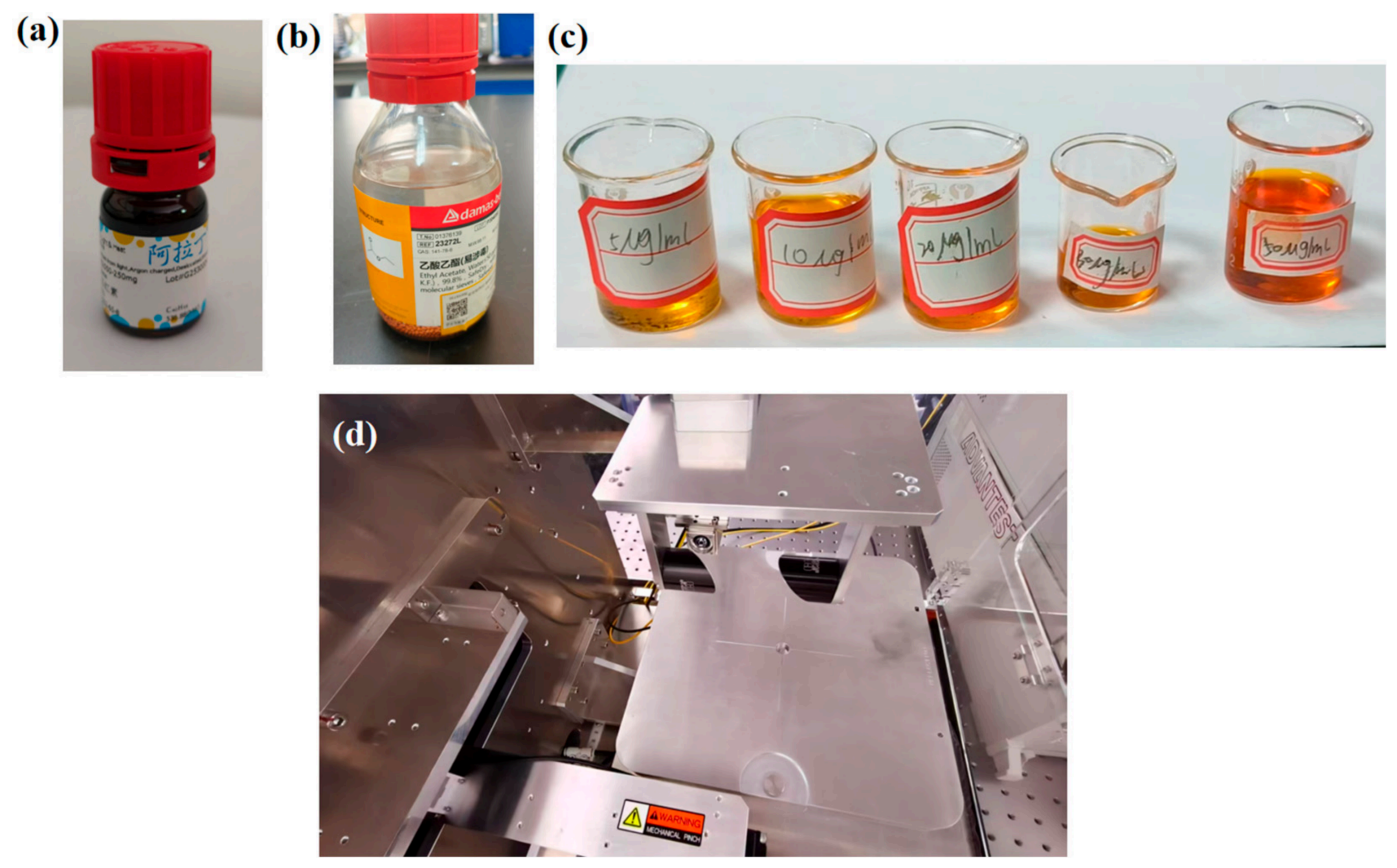
(**a**) Lycopene (C_40_H_56_), red crystalline powder; (**b**) ethyl acetate (C_4_H_8_O_2_); (**c**) lycopene solutions with different concentrations; (**d**) terahertz measurement setup.

Lycopene (C_40_H_56_), a red crystalline powder with purities of ≥95%, and ethyl acetate (C_4_H_8_O_2_), with purities of ≥99.8%, were purchased from Shanghai Aladdin Biochemical Technology Co., Ltd. (Shanghai, China). Lycopene solutions with concentrations of 5 μg/mL, 10 μg/mL, 20 μg/mL and 30 μg/mL were prepared. The lycopene solutions were drop-cast onto the sensor chip. After air-drying, the samples were characterized using the terahertz time-domain spectroscope (THz-TDS) system, as illustrated in Figure 6.

**Figure 7:**
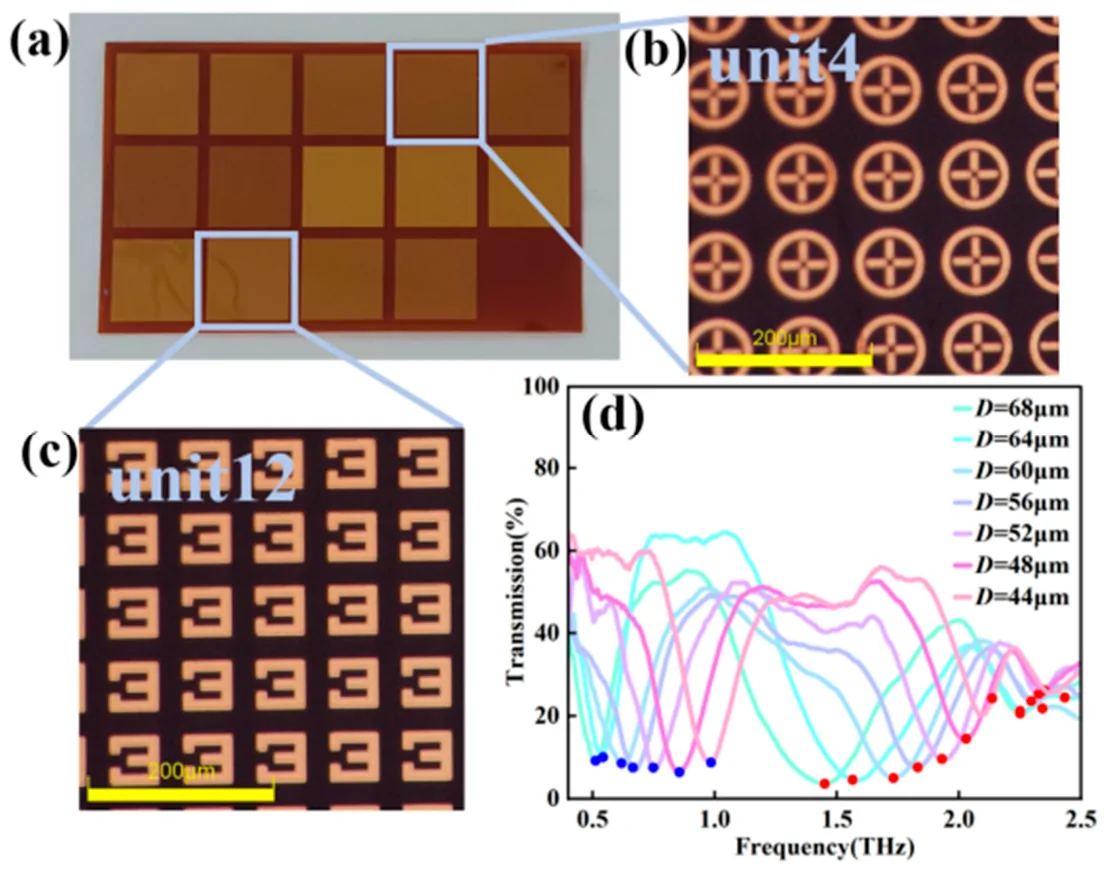
(**a**) Optical photograph of a dual-design array-element-integrated metasurface terahertz sensing chip; (**b**) local magnified optical image of cross-shaped metasurface; (**c**) E-shaped metasurface local magnified optical image; (**d**) partial transmission spectrum of a dual-design array-element-integrated metasurface terahertz sensing chip.

**Figure 8:**
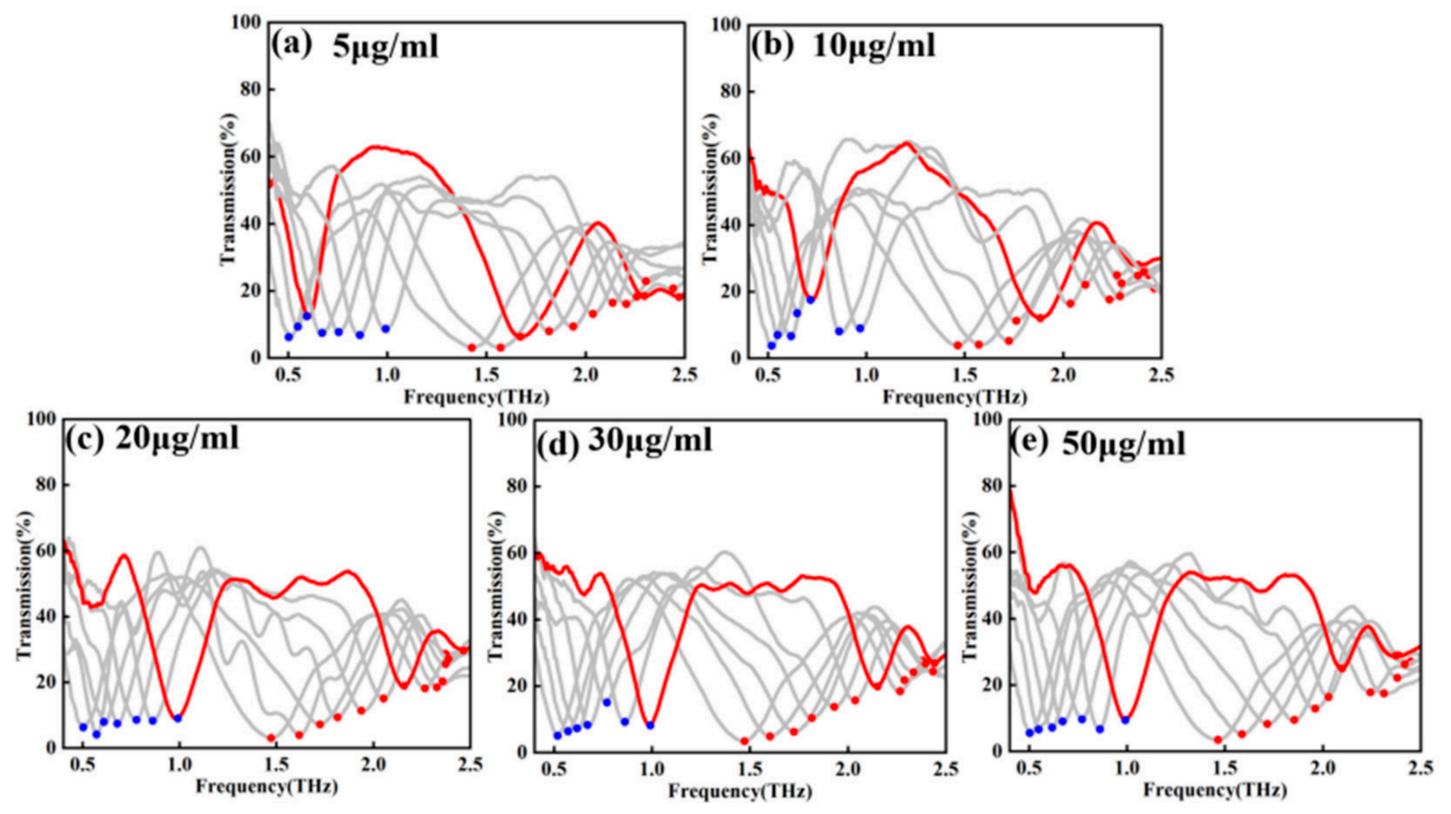
Terahertz wave transmission spectra of lycopene concentrations of (**a**) 5 µg/mL, (**b**) 10 µg/mL, (**c**) 20 µg/mL, (**d**) 30 µg/mL, and (**e**) 50 µg/mL.

**Figure 9:**
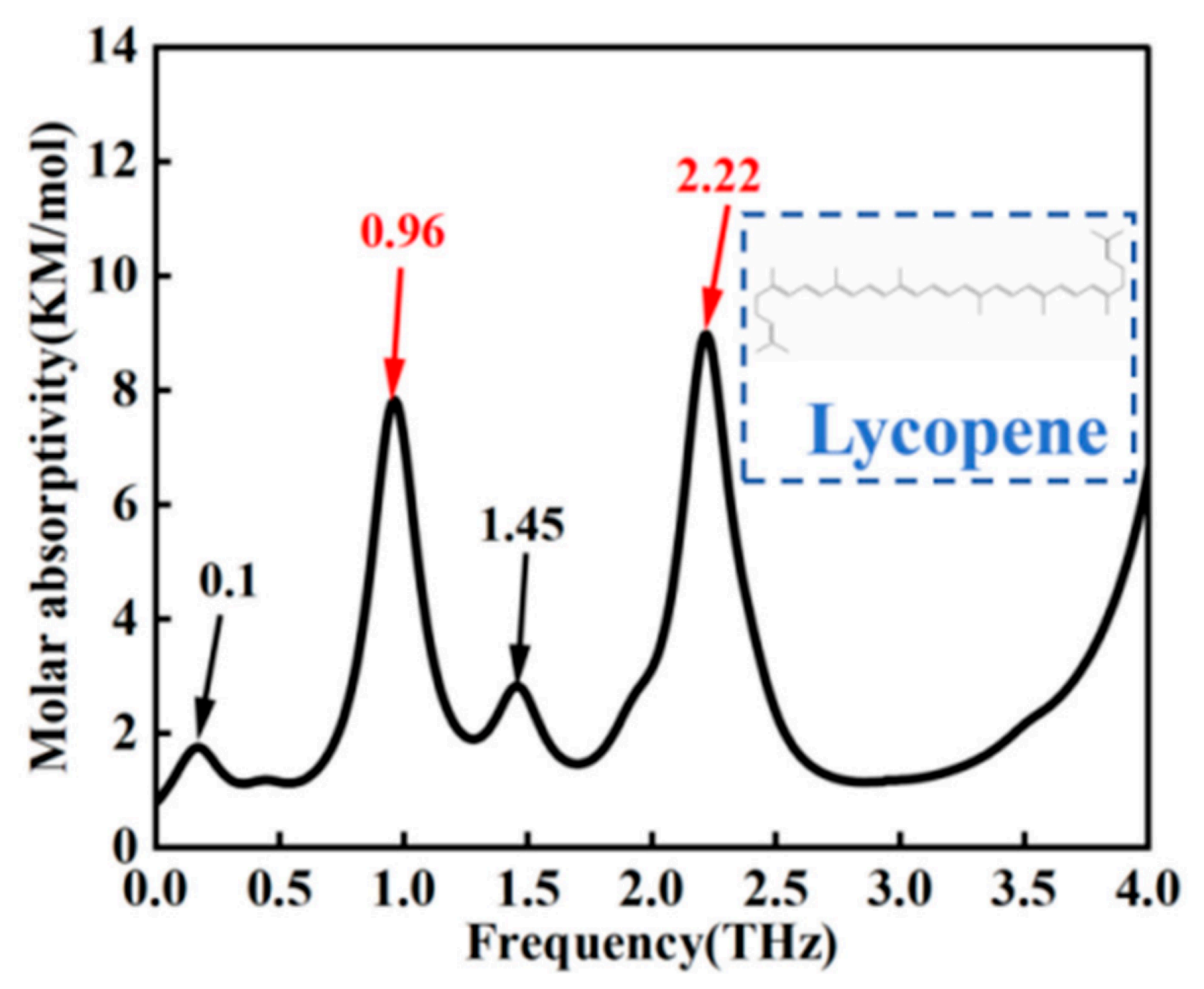
The calculated terahertz response curve of lycopene.

Figure 7a shows an optical photograph of a dual-design array-element-integrated metasurface terahertz sensing chip, consisting of 14 metasurface arrays. The optical image of the fourth element (crosshair-shaped) is locally magnified to obtain the image shown in Figure 7b. Figure 7c presents the locally magnified optical image of the twelfth element (E-shaped). Figure 6d displays a partial transmission spectrum of the sensing chip, with the lowest point of the transmission peak marked as the resonance point. The blue point is the resonance point of the first envelope curve, and the red point is the resonance point of the second envelope curve. In subsequent analysis, a two-stage envelope curve is used for analysis. Figure 8a–e show the terahertz wave response of the metasurface after coating with lycopene solutions at concentrations of 5 µg/mL, 10 µg/mL, 20 µg/mL, 30 µg/mL, and 50 µg/mL. At concentrations of 5 µg/mL and 10 µg/mL, the first envelope curve shows a marked blue point shift. At concentrations of 20 µg/mL, 30 µg/mL, and 50 µg/mL, the range of the second envelope curve shows a marked red point shift. The tested results indicate that the lower concentration range affects the response of the first envelope segment, while the higher concentration range acts on the second envelope segment. To establish the correspondence between the response frequency of the metasurface and the molecular structure, Gaussian software (GaussView 6.0) was used to calculate the four terahertz absorption peaks of lycopene, which are 0.1 THz, 0.96 THz, 1.45 THz, and 2.22 THz, respectively, as shown in Figure 9. The above-envelope curve measurement results include fingerprint characteristic peaks at 0.96 THz and 2.22 THz, proving that the sensor has a fingerprint detection function.

**Figure 10:**
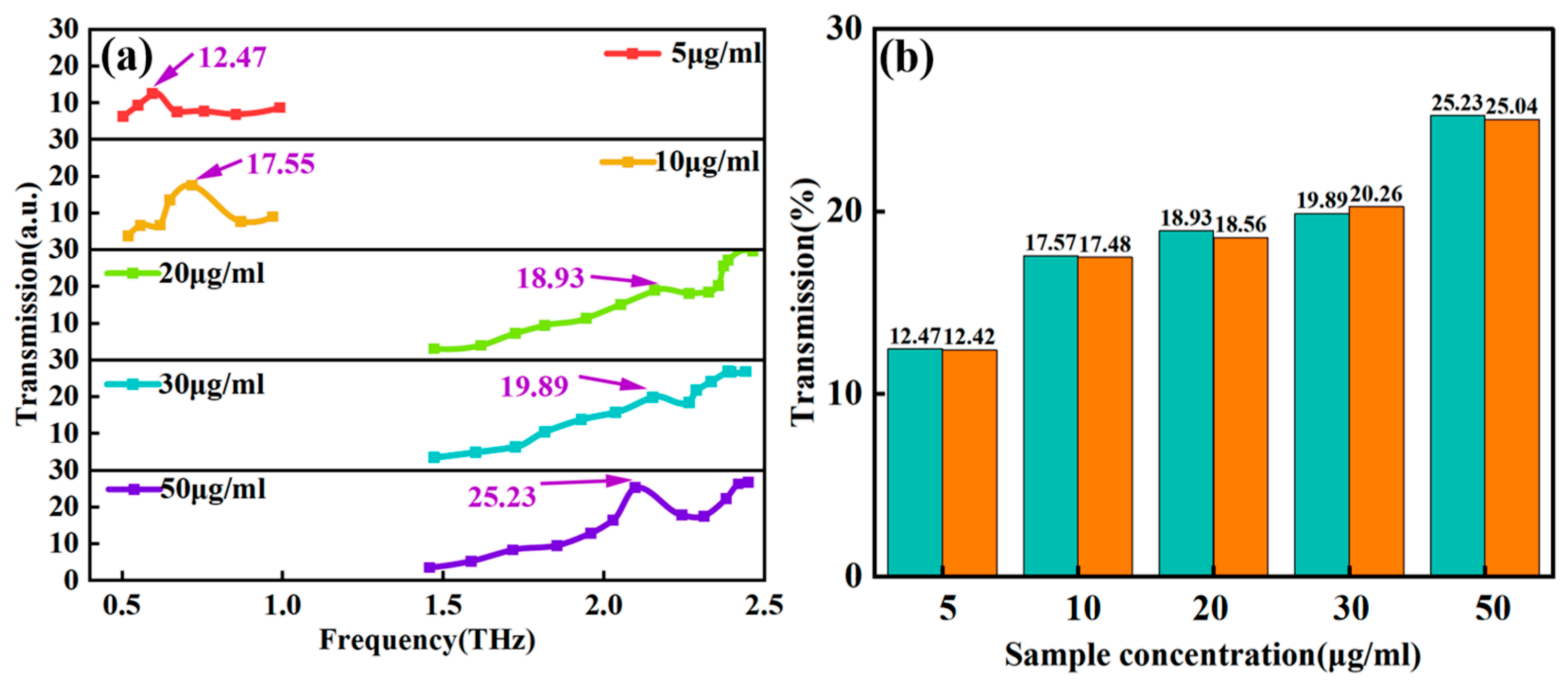
The lycopene concentrations of 5 μg/mL, 10 μg/mL, 20 μg/mL, 30 μg/mL, and 50 μg/mL: (**a**) terahertz detection envelope curve; (**b**) error bars analysis.

Figure 10a shows the envelope resonance point envelope curves of the first or second segment at different concentrations. When the concentration of lycopene is 5 μg/mL and 10 μg/mL, the corresponding peak values of the first envelope curve are 12.47% and 17.55%, respectively. The peak values of the third envelope curve corresponding to lycopene concentrations of 20 μg/mL, 30 μg/mL, and 50 μg/mL were 18.93%, 19.89%, and 25.23%, respectively. As the concentration of lycopene solution increases, the peak values of both envelope curves increase. To clearly demonstrate the relationship between the two envelope curves and concentration, Figure 10b presents the bar chart of error analysis at various lycopene concentrations. The figure shows only a slight deviation between the measured and true values. The sensitivity of the first envelope curve can be calculated as 10.44 × 10^−3^ (a.u./(μg/mL)), and the sensitivity of the second envelope curve is 2.67 × 10^−3^ (a.u./(μg/mL)). Further verification was conducted on the quantitative detection capability of the terahertz envelope detection sensor for lycopene concentration, and the detection limit of the chip for lycopene was found to be 5 μg/mL. In summary, the terahertz envelope detection sensing chip not only maintains stable characteristic fingerprint spectra, but also achieves sensitive and reliable detection of target concentration through linear changes in transmission peaks, which has potential application value in the field of biochemical molecular sensing.

**Figure 11:**
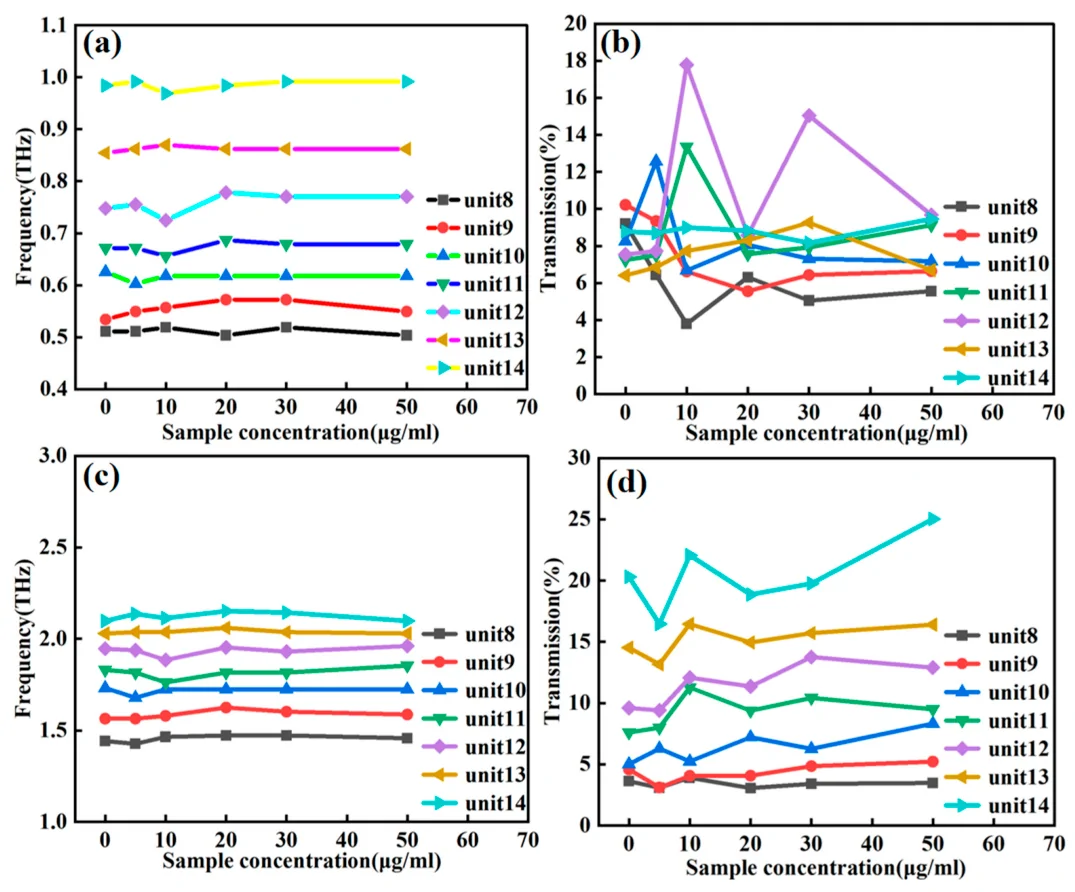
(**a**) Frequency shift and (**b**) amplitude variation in the first envelope; (**c**) frequency shift and (**d**) amplitude variation in the partial second envelope for the Unit 8–14 metasurface under lycopene solution concentrations of 5, 10, 20, 30, and 50 μg/mL.

Figure 11 shows the frequency shift and amplitude curves of unit8 to unit14 of the metasurface array at lycopene solution concentrations of 5 μg/mL, 10 μg/mL, 20 μg/mL, 30 μg/mL, and 50 μg/mL. Figure 11a presents the frequency shift curves of the first segment envelope. It can be observed that each metasurface unit exhibits a slight redshift as the concentration increases, with values staying within a certain range, demonstrating reasonable robustness. Figure 11b plots the amplitude variation curves of the first segment envelope. At a lycopene concentration of 5 μg/mL, unit10 imposes the strongest influence on the transmittance of terahertz waves. Jump changes in the terahertz transmittance of unit12 occur at lycopene concentrations of 10 μg/mL and 30 μg/mL. Figure 11c displays the frequency shift curves for the partial envelope of the second segment, which resemble those of the first segment and fluctuate mildly within a bounded range. Figure 11d shows the amplitude variation curves for the partial envelope of the second segment. The terahertz transmittance of each metasurface unit increases with rising concentration. A comparison of sensing performance between recently reported metasurfaces and the present work is presented in Table 1, which reveals a significant improvement in sensing sensitivity achieved herein.

## 4. Conclusions

To sum up, we designed a terahertz metasurface sensing chip integrating a composite array of crosshair-shaped and E-shaped unit structures, capable of achieving envelope-based biochemical sensing detection over a broad frequency range of 0.50–2.50 THz. Through multipole scattering analysis and near-field distribution studies, it reveals the LSPR mechanism dominated by electric dipoles (EDs) in the crosshair-shaped metasurface, as well as the EIT effect induced by multimode coupling of EDs, electric quadrupoles (EQs), and magnetic dipoles (MDs) in the E-shaped metasurface. With lycopene as the target analyte, the chip exhibits significant responses near its characteristic absorption peaks, demonstrating a strong linear relationship between transmittance peak intensity and sample concentration. The highest sensitivity reaches 10.44 × 10^−3^ a.u./(μg/mL), and the lowest experimentally detected concentration is 5 μg/mL. The experimental results indicate that this chip demonstrates excellent performance in wide-spectrum fingerprint identification and trace detection, and holds promise for application in highly sensitive sensing of other biochemical molecules.

## Figures and Tables

**Table 1:** Sensing performance comparison of recently reported metasurfaces with this work.

Reference	Mechanism	Material	Operating Frequency Band	Sensitivity	Analyte
[19]	QBIC	Copper	1–2.8 THz	500 GHz/RIU	Glu and Gln
[20]	data	AL	-	56 GHz/RIU and 36 GHz/RIU	cis-AB, trans-AB and Lactose
[21]	Fano	Gold	0.18–1.20 THz	-	L-tyrosine + bovine hemoglobins
[22]	-	SWCNT	0.65–1.09 THz	0.68 GHz/nmol and 0.81 GHz/nmol	Benzoic acid and Leucine
[23]	EIT	Gold	0.89 THz, 1.56 THz	280.8 GHz/RIU and 201.6GHz/RIU	-
[24]	EIT-BIC	silicon	2.16 THz, 2.42 THz	457.6 GHz/RIU and 607.5 GHz/RIU	-
This work	LSPR + EIT	Gold	0.50–2.50 THz	10.44 × 10^−3^ a.u./(μg/mL)	Lycopene

## Data Availability

The data that support the findings of this study are available from the corresponding authors upon reasonable request.
